# Two and three-dimensional CT mapping of the sustentacular fragment in intra-articular calcaneal fractures

**DOI:** 10.1038/s41598-022-24916-w

**Published:** 2022-11-28

**Authors:** Guang Shi, Zhao Lin, Xun Liao, Wei Liu, Xiyu Cai

**Affiliations:** Department of Orthopedics, The Fifth Affiliated Hospital of Sun Yat-Sen University, Zhuhai, 519000 Guangdong Province China

**Keywords:** Anatomy, Engineering, Mathematics and computing

## Abstract

The sustentaculum tali are tightly bound to the talus by the interosseous and deltoid ligament complex and have been considered a ‘‘constant fragment”. Yet there is a dearth of study on the anatomical patterns of the sustentacular segment. Consequently, this study is designated with the purpose of defining the prevalence and displacement of sustentacular fractures in intra-articular calcaneal fractures (ICFs) applying computed tomography (CT) mapping in both two-dimensional (2D) and three-dimensional (3D) conditions. From January 2019 to December 2020, the CT images of sixty-seven patients with eighty-one ICFs were retrospectively evaluated, besides, basic patient demographics and mechanisms of injury were documented. And the prevalence of sustentacular fractures was characterized in the sagittal or coronal CT planes. The subluxation, angulation, and translation of the portion of the sustentacular bone were noted. By decreasing rebuilt fracture fragments to suit a model of the sustentaculum tali, a 3D map was generated. Overall, the sustentacular fracture in 21 (25.9%) of the 81 ICFs, 15 (71.4%) were nondisplaced, 6 (28.6%) were displaced, and mean coronal angulation was 21.9°, and no comminuted. The relationship between sustentaculum tali and the talus was anatomically aligned in 71 (87.7%), and subluxation in 10 (12.3%). According to the research, 3D mapping demonstrated that most fracture lines start from the anterior of the sustentaculum tali, extending obliquely to the sulcus of the flexor halluces longus tendon. Moreover, this study provides a detailed description (displacement or articular dislocation) of the frequency of sustentacular fragments in ICFs. The finding disproves the ‘‘constant’’ theory of the sustentacular fragments, due to the fact that comminuted fracture of sustentaculum tali was rare. And the expertise of these fracture patterns may affect the progress of fixation concepts and surgical approaches. Moreover, we further speculated that the displacement of the sustentacular fragment was considerably more probable to emerge in the higher-order Sanders classification. Nevertheless, bigger sample size is required to further validate this position.

## Introduction

The sustentaculum tali, also identified as the talar brace, is a horizontal, biomechanically solid process located on the medial and distal portion of the calcaneus^1^, and is tightly combined with the talus via the interosseous talocalcaneal ligaments, spring ligament, and deltoid ligaments, resulting in an anatomical consistency and is referred to as the ‘‘constant’’ fragment^2–6^. Advancements in standard imaging studies and internal fixation techniques have improved the surgical treatment of ICFs^7–9^. This sustentacular fragment was essential for restoring the subtalar joint during surgical fixation. Displaced ICFs have been open reduction and internal fixation through a variety of surgical incisions^4,10,11^. The most typical lateral surgical approach and minimally invasive sinus tarsal approach, but with restricted access to the interior of the calcaneus and visualization of the medial sustentaculum tali^12^. Furthermore, one of the critical factors in fracture reduction and screw fixation was the sustentaculum tali. Thus, a greater visual comprehension of the sustentacular fragment's anatomical structure was required.

Although the concept of anatomic stability of the sustentaculum tali in ICFs has lasted for numerous years, and it was not until 2013 that a deeper understanding was obtained^13^. With the advent of digital medicine, it is now possible to detect displacement of sustentacular fractures in both 2D and 3D environments.

This study aims to access the prevalence and displacement of sustentacular fractures in patients of the ICFs using 2D and 3D mapping techniques, as well as to explore the correlation between the incidence of sustentacular fragments and Sanders classification. We hypothesized that fracture mapping would reveal more comprehensive sustentacular fragment patterns than prior X-ray and CT reports.

## Materials and methods

### Subjects

This investigation of the fifth affiliated Hospital of Sun Yat-sen University was approved by the ethics committee. All procedures were carried out in accordance with the pertinent norms and regulations. We conducted a retrospective review of patients from January 2019 to December 2020 with ICFs. All patients underwent CT scans (Siemens, Berlin, Germany), and the image was preserved as Digital Imaging and Communications in Medicine (DICOM) files. Medical records were mined for primary demographic data (including age, gender, classification, side, and mechanism of injury).

**Figure 1:**
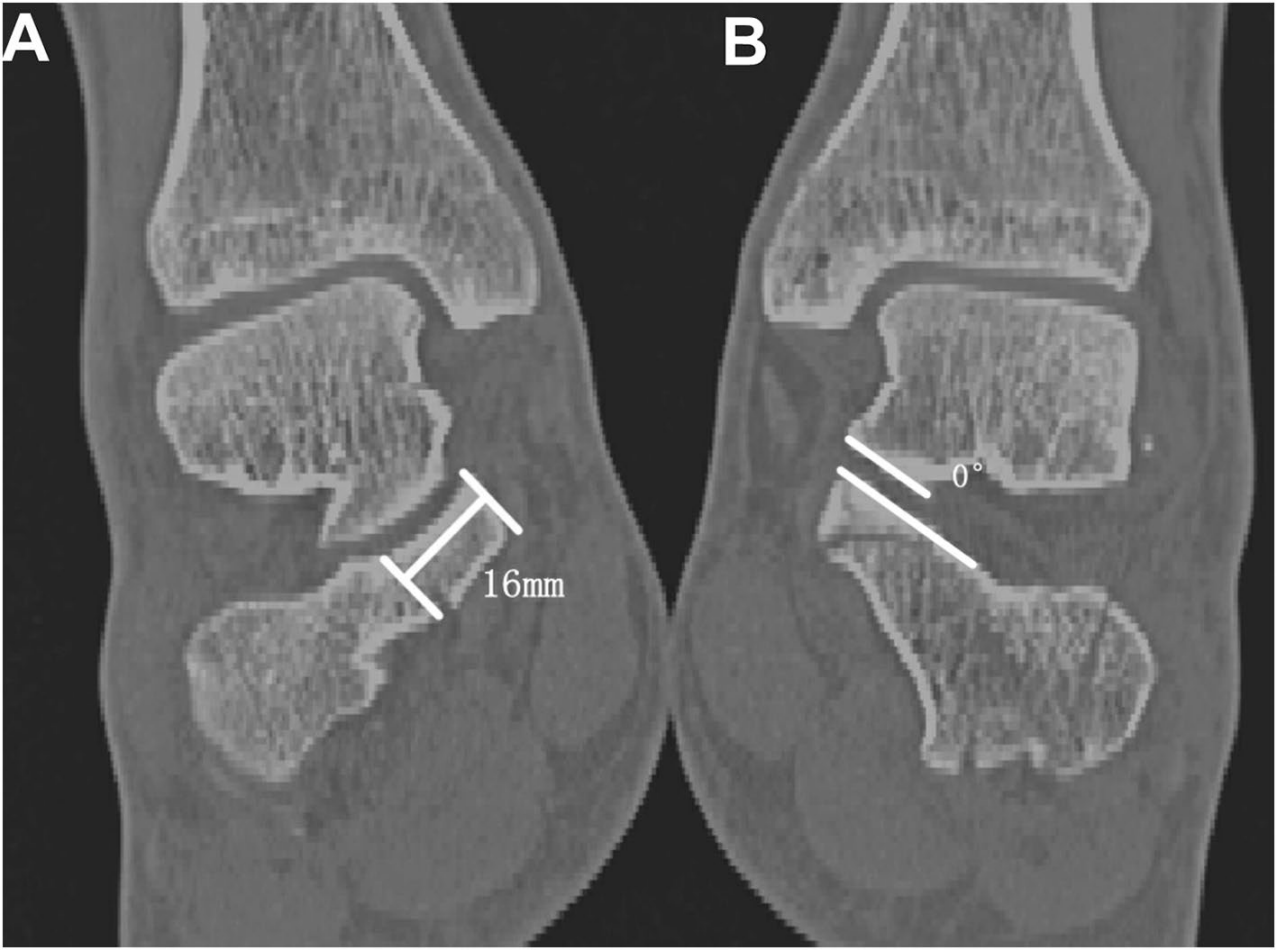
The method used for 2-D CT mapping of sustentacular fracture. (**A**) Coronal CT image showing the sustentacular fracture. (**B**) Assessment of angulation in the coronal.

**Figure 2:**
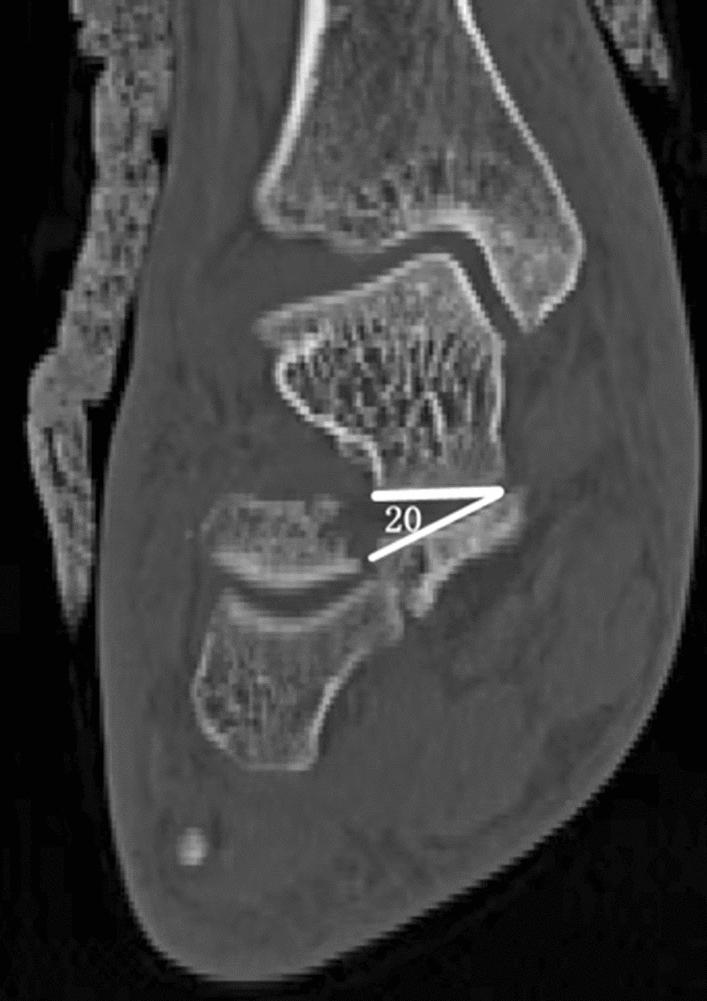
Coronal CT showings the angulation of the sustentaculum fragment.

Raw CT data were transferred into Mimics 21.0 software (Materialise) and were reviewed and independently assessed by two professional radiologists*.* Previous studies have indicated that the sustentaculum tali comprise approximately one-third of the transverse width of the calcaneal, with an average length of 13 mm in the transverse plane of the calcaneus^14^. Consequently, we designated the sustentaculum tali as the 13-mm-long extension of the most medial prominence. And CT scans of non-displaced calcaneal fractures served as controls for determining the normal limits of measuring parameters and the ideal CT cuts for evaluating various metrics (Fig. 1). By analyzing the prevalence and displacement of sustentacular pieces in coronal and sagittal planes using measurement angulation, subtalar dislocation was determined. Any angulation exceeding 10° and translation of more than 2 mm, and the displacement diagnostic has been considered. Angulation > 5° of articular surfaces were considered subluxation. Sustentacular fracture, displacement, and dislocation of the talus sustentaculum were documented^2^ (Fig. 2).

### Fracture mapping

The method of fracture mapping was described by Cole et al.^15^. Sustentacular fragments were multiplanar reconstructed in Mimics and were virtually reduced. The data was then imported into the 3-Matic 13.0 program (Materialise), and the rebuilt pieces were rotated, standardized, and horizontally inverted so that they corresponded most closely to a 3D model of the sustentaculum tali. Smooth curves were delineated precisely on the template surface to reproduce the fracture line distribution of each sustentaculum tali in 3-Matic. Subsequently, all sustentaculum tali fracture lines were evaluated.

### Statistical methods

Measurements of the patient and sustentacular fragment baseline parameters are summarized as the mean, standard deviation, or percentage. Patterns of fragmentation and fragment and displacement were analyzed qualitatively. Qualitative data is presented as the number (percentage) and quantitative data is expressed as the mean (SD) using SPSSVs. 21.

### Ethics approval and consent to participate

This study was approved by the local ethics committee (the Fifth affiliated Hospital of Sun Yat-sen University). Data were analyzed retrospectively; informed consent was obtained from all participants.

## Results

### Patient characteristics

Over the study period (from January 2019 to December 2020), we retrospectively reviewed records of 67 patients with 81 calcaneal fractures that matched the inclusion criteria. Containing 26 (38.8%) left calcaneal fractures, 27 (40.30%) right calcaneal fractures, and 14 (20.90%) cases with bilateral fractures, including 21 cases of sustentacular fractures. Moreover, 21 sustentacular fractures included 9 (42.9%) left sustentacular fractures, 11 (52.4%) right sustentacular fractures, and 1 (4.7%) case with bilateral fractures. The average age was 44.50 (SD 11.80). 17 were male (81.0%), while 4 were female (19.0%). 19 (90.5%) were the result of falling accidents, whereas 2 (9.5%) were the result of motor accidents.

### Coronal maps

A sustentacular fracture was present in 21 (25.9%) of the 81 fractures; type III and type IV accounted for 16 (76.2%). 15 (71.4%) of the sustentacular fragment were nondisplaced, 6 (28.6%) were displaced, of which displaced Sanders IV accounted for 5 (83%), with no comminuted fractures. Substantially more sustentacular fragment displacement was observed in higher-order Sanders classifications. The link between the sustentaculum tali and the talus was anatomically aligned in 71 (87.7%) and subluxation occurred in 10 (12.3%), and the average coronal angle was 21.9°. Moreover, as can be seen in Table 1, we evaluated the patient characteristics of those with and without sustentacular involvement: encompassing injury process, fracture morphology, and injuries related to calcaneal fractures. All sustentacular fractures are accompanied by fractures at other locations (such as tibiofibular fractures, vertebral fractures, talus fractures, fracture of the distal radius, acetabulum fracture, scaphoid bone fracture, patellar fractures, pilon fractures, etc.), and sustentacular fragments are frequently associated with falls.

**Table 1:** Patient characteristics (including mechanism of injury, fracture morphology and associated injuries) of calcaneal fractures with and without sustentacular involvement.

Characteristics	None sustentacular fractures (n = 56)	Sustentacular fractures(n = 21)
*Patient demographics*		
Fracture mechanism, n (%)		
Falling accidents	51 (91.1)	19 (90.5)
Traffic accident	5 (8.9)	2 (9.5)
Fracture patterns, n (%)		
Simple	3 (5.4)	21 (100)
Comminution	53 (94.6)	No
Concomitant fractures, n (%)		
Fracture	29 (51.8)	21 (100)
No	28 (48.2)	No

### 3D maps

**Figure 3:**
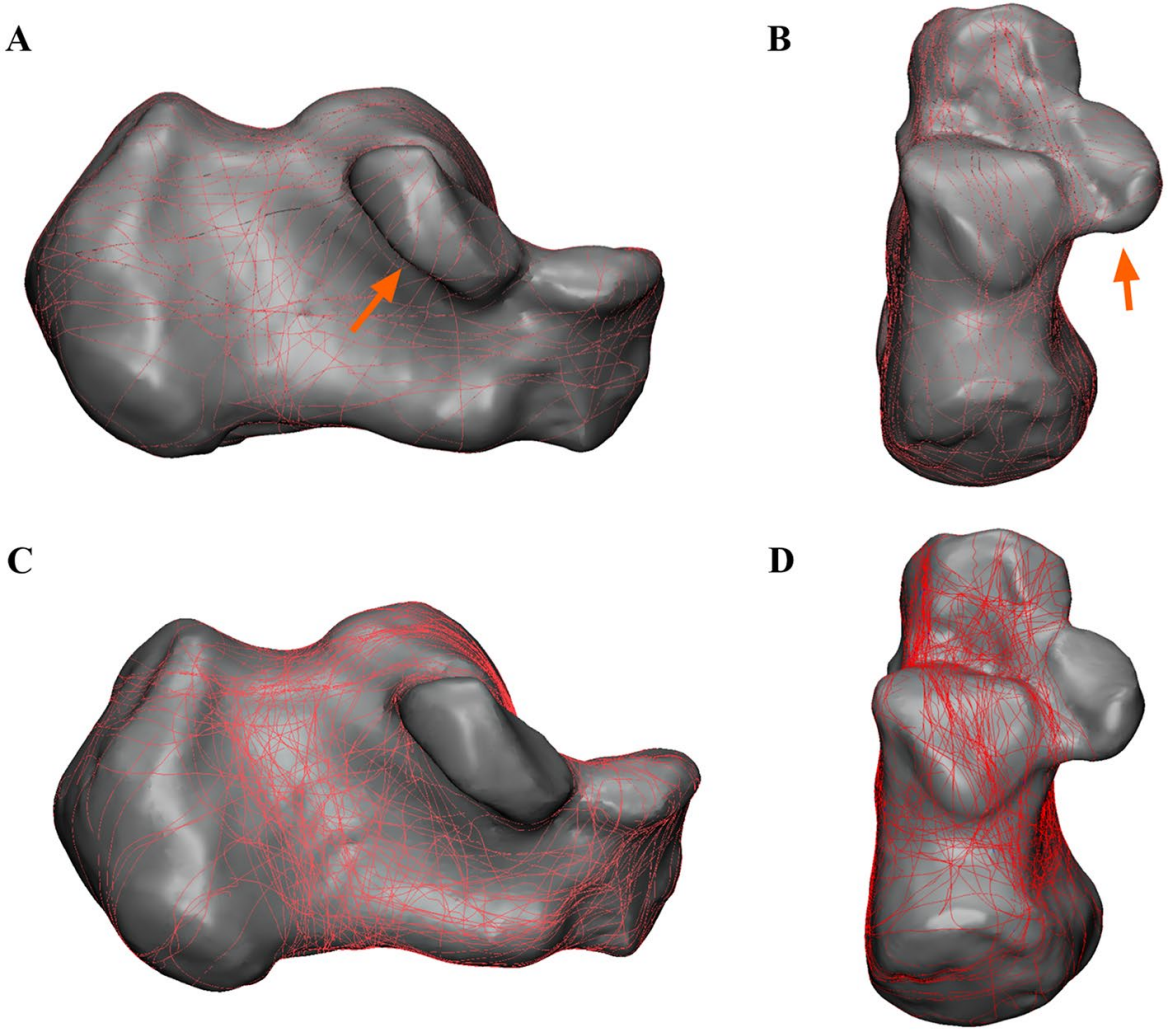
The method used for 3D CT mapping of sustentacular fracture. (**A**, **B**) Representative views of the 3D map of the 21 sustentacular fracture lines. (**C**, **D**) Representative views of the 60 patients without sustentacular fractures.

**Figure 4:**
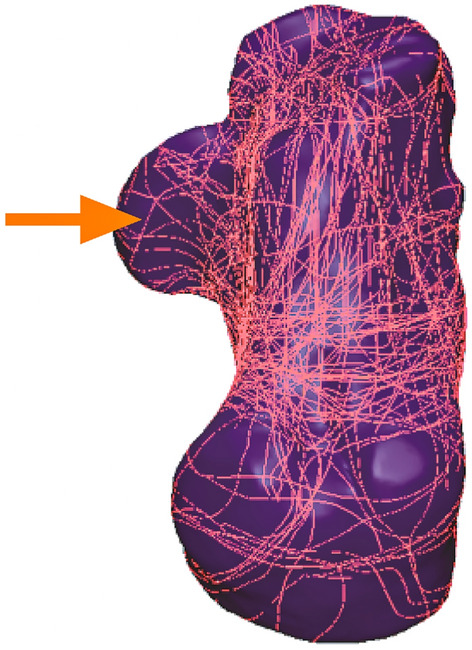
The method used for 3D CT mapping of sustentacular fracture. Representative views of the 3D map of the 81 calcaneal fracture lines.

In each of the 67 patients, the fracture line was grouped on a typical calcaneal template, with separate fracture maps of the 21 patients with and the 60 patients without sustentacular fractures (Fig. 3A–D). In the sustentaculum tali, the majority of the fracture lines begin at the front of the sustentaculum tali, extending obliquely to the sulcus of the flexor halluces longus tendon (Fig. 4).

## Discussion

This study aims to determine the frequency and displacement of sustentacular fragments in patients with ICFs by fracture mapping technology and to determine that the sustentacular fragment is not a "constant" fragment. We also sought to evaluate the association of sustentacular fractures with sanders’ classification. Our findings revealed that in ICFs, the sustentacular fragment was not a "constant" fragment and the middle facet’s position was not always stable. There appears to be a link between the risk of displacement of sustentacular fragments and Sanders's classification.

Heger et al.^6^ a study of calcaneal fractures evaluated the displacement of sustentacular fragments in 18 of 25 fractures (72%). They assume that the sustentacular fragments were anatomically consistent with the talus. Subsequently, Gilmer et al.^16^ in the CT study of 32 calcaneal fractures, presume that the deltoid and interosseous talocalcaneal ligament maintained the position of the fragments of the sustentaculum, anatomically aligned with the talus, and never displaced. To our knowledge, Berberianetal et al., in a coronal CT study of 100 displaced ICFs, revealed more specific data on the occurrence of excessive angulation rates, translation, and fracture diastasis of the sustentacular fragments^13^. They also observed an increase in the prevalence of high-grade Sanders subtype-B and C classification. In 2014, Gitajn et al. included 212 patients with calcaneal fractures and reported in detail the incidence, displacement, and anatomical alignment of sustentacular fractures^2^. Our findings concur with Berberianet al. and Gitajn et al. in that patients with intra-articular calcaneal fractures challenge the assumption of anatomic constancy for sustentacular fragments. The general 2D and 3D sustentaculum tali revealed the prevalence and displacement of sustentacular fractures, as well as the superimposition of the fracture line, illuminating the fracture pattern of the sustentaculum tali, and providing more detailed information than conventional CT.

The sustentaculum tali are essential in maintaining the internal foot column, so conservative treatment may not be appropriate for displaced sustentacular fractures. Although the extensile lateral approach and tarsal sinus approach are most commonly employed for the operational treatment of calcaneal fractures^17–20^, the tarsal sinus method is gaining popularity^17–20^. One downside of this isolated lateral incision is that the sustentacular fragments cannot be managed directly, and the visualization of the sustentacular fragments is limited. Besides, the operation can only be accomplished through the surgeon's indirect experience to perform the reduction. And the flexor hallucis longus tendon may insert into the fracture line, resulting in a reduction complicated and leading to non-union^21^. In addition, sustentacular fractures are difficult to diagnose on lateral X-rays, hence misdiagnosis is common. Misdiagnosis and mistreatment of the sustentacular fractures can lead to severe issues, posttraumatic subtalar joint arthritis, fracture non-union, chronic impingement of the flexor hallucis longus tendon, paraesthesia of the medial plantar nerve, persistent pain and swelling of the medial hindfoot, progressive pes-planovalgus deformity or tarsal tunnel syndrome^22,23^. In this study, the 3D mapping of the sustentaculum tali revealed that the fracture lines start from the anterior of the sustentaculum tali, extending obliquely to the sulcus of the flexor halluces longus tendon. Consequently, the complexity of surgical reduction necessitated the consideration of introducing the flexor hallucis longus tendon into the fracture lines.

Understanding the fracture pattern of the sustentacular bone can aid in determining the optimal surgical strategy. The medial approach over the sustentaculum has been described by Zwipp et al. back in 2004 and detailed further by Durr et al.^1^. Additionally, Della et al.^24^ in a CT study of 15 sustentacular fractures and indicate that approximately half of these fractures affected the articular surface of the subtalar joint. An isolated medial approach can anatomically reduce the sustentacular fragment, yet visualization and anatomic reduction of a depressed posterior-facet fracture are not feasible^4,25^. There appears to be a correlation between the risk of sustentacular fragment displacement and our findings and Sanders’s classification. The displacement of the sustentacular fragments occurs mainly in Sanders Type IV. Zwipp et al.^10^ have also proposed the reduction of the tilted sustentacular fragment as the first step for calcaneal fracture reduction in complex fracture patterns. Accordingly, it seems reasonable to attempt a medial approach for an isolated fracture of the sustentaculum tali. Yet, medial and lateral treatments may be more appropriate for complex ICFs with sustentacular fractures.

The sustentaculum tali are predominantly formed by cortical bone, with high trabecular bone density, which provides solid medial support throughout Surgical fixation^24,26^. In this study, 6 (28.6%) were displaced, the mean coronal angulation was 21.9°, and the subluxation was 10 (12.3%). This may be related to the internal structure of the sustentaculum tali, which lacks a comminution area. Furthermore, the fracture patterns revealed in our study may enhance the understanding of injury associated with the sustentaculum tali. In our research, 6 (28.6%) were displaced, of which displaced Sanders IV accounted for 5 (83%), with a subluxation in 10 (12.3%). Our results indicate that the surrounding ligaments were probably to have been injured, challenging the notion that the fragments are anatomically consistent and integrated with the talus via the interosseous and deltoid ligaments. Hence, when preoperative CT scans reveal displacement or subluxation of the sustentacular fractures, we advise further MRI evaluation to establish the damage condition of the interosseous and deltoid ligaments.

Nevertheless, there are some drawbacks to this study. Firstly, evaluation was limited to CT data and no clear assessment of the functional impact or related consequences of these imaging results. Second, patients who had or lacked adequate CT imaging were excluded, which may affect the accuracy of the results. Third, the number of patients is moderately low, and more patients may result in more accurate results. In this study, 6 (28.6%) were displaced, of which displaced Sanders IV accounted for 5 (83%). Thus, we hypothesize that the sustentacular fragment was considerably more likely to be displaced in the Sanders classifications with a higher order. Nonetheless, due to the tiny size of the sample, we could not perform correlation analysis; a larger sample size is needed to validate this view further. Finally, postoperative CT scans were not obtained, and the quality of calcaneal reduction in patients with sustentacular fracture compromise was not identified.

## Conclusion

Our study provides a comprehensive description (displacement or articular dislocation) of the frequency of sustentacular fragments in ICFs. The finding disproves the theory that sustentacular fragments are “constant”. In addition, knowledge of these fracture patterns can affect the development of fixation concepts and surgical approaches.

## Data Availability

The datasets generated during and/or analyzed during the current study are available from the corresponding author on reasonable request.

## Abbreviations

CT: Computed tomography
2D: Two-dimensional
3D: Three-dimensional
CFs: Intra-articular calcaneal fractures
